# Supplementary material for: Sexually dimorphic architecture and function of a mechanosensory circuit in C. elegans
Source: Nat Commun. 2022 Nov 11;13:6825. doi: 10.1038/s41467-022-34661-3 (PMC9652301; doi:10.1038/s41467-022-34661-3)
Supplement: Supplementary file 1 — Supplementary Information [file 41467_2022_34661_MOESM1_ESM.pdf]

SUPPLEMNETAL INFORMATION

Sexually dimorphic architecture and function of a mechanosensory circuit in *C. elegans*

This file includes ten supplementary figures and a supplementary table.

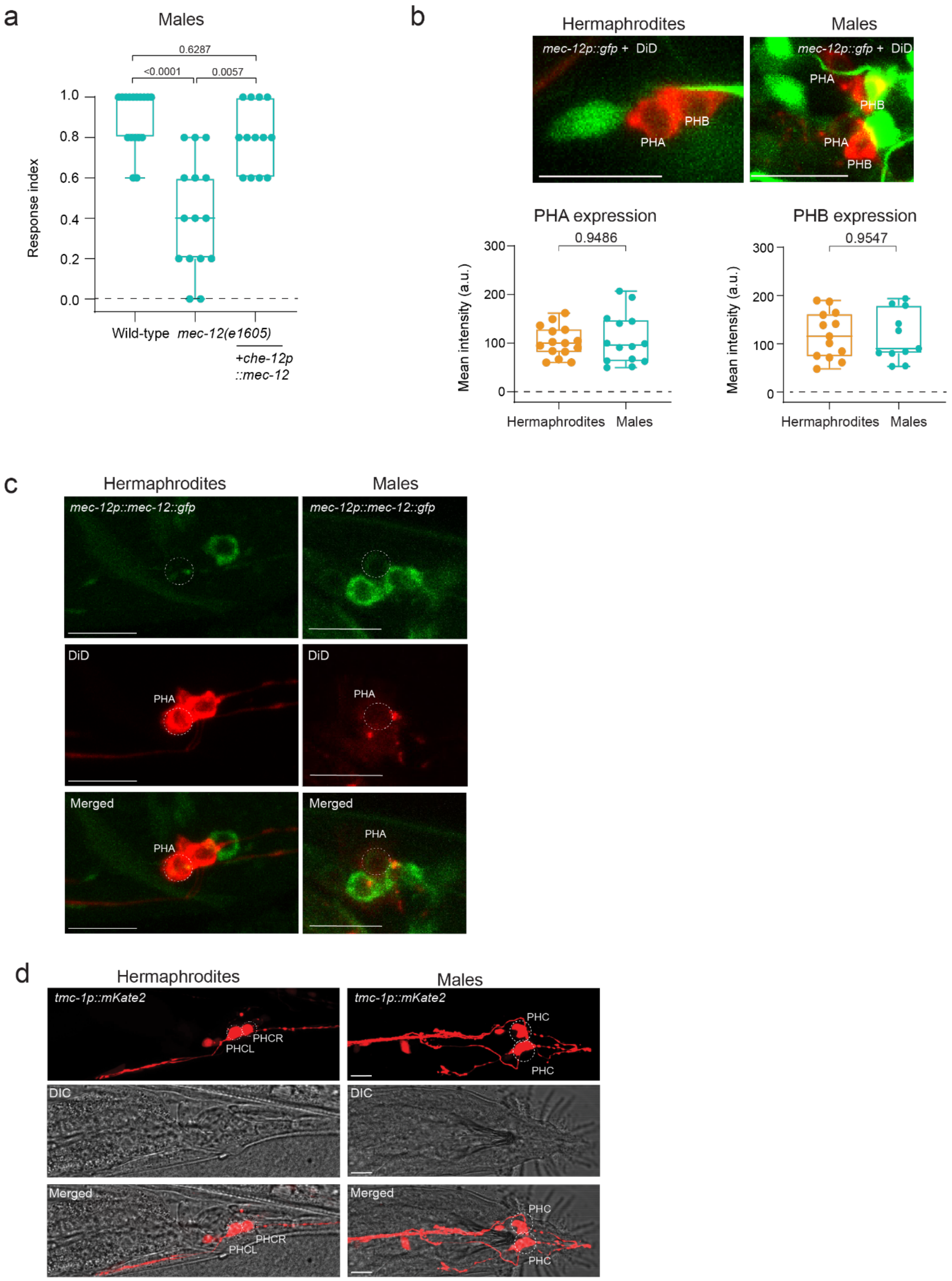

Supplementary Fig. 1: Expression pattern of *mec-12* and *tmc-1* in tail sensory neurons

a Tail-touch responses of wild-type (n=17), *mec-12(e1605)* (n=15) and *mec-12(e1605);che-12p::mec-12* (n=13) males. We performed a Kruskal-Wallis test followed by a Dunn's multiple

comparison test. **b** Representative confocal micrographs of a transcriptional *mec-12p::gfp* reporter in hermaphrodites and males. Scale bars are 10 µm. Quantification and comparison of *mec-12p::GFP* expression levels in PHA and PHB. a.u., arbitrary units. Hermaphrodites, PHA: n=15, males, PHA: n=14, hermaphrodites, PHB: n=13, males, PHB: n=11. We performed a two-sided Mann-Whitney test for each comparison. PHA and PHB were identified using DiD staining. **c** *mec-12p::mec-12::gfp* protein reporter in hermaphrodites and males in PHA. PHA was identified using DiD staining. Scale bars are 10 µm. n=10 animals per group were examined for *mec-12* expression. **d** Representative confocal micrographs of *tmc-1p::mKate2* in both sexes. Scale bars are 10 µm. PHC was identified using the *otIs520* transgene[1]. n=10 animals were examined for *tmc-1* expression. All Bar graphs are a box-and-whiskers type of graph, min to max showing all points. The vertical bars represent the median.

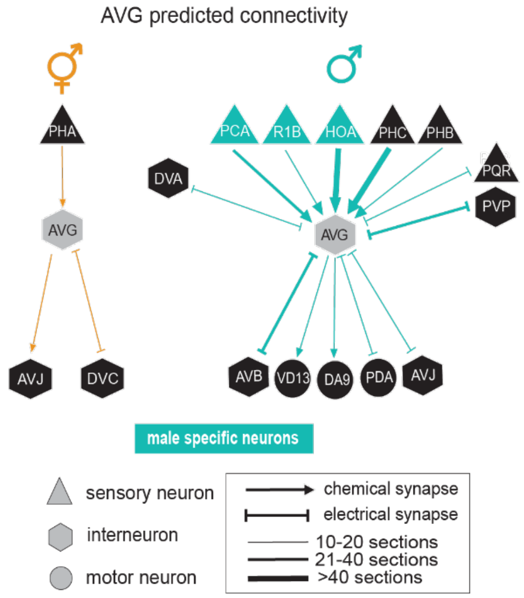

Supplementary Fig. 2: The sex-shared interneuron AVG receives many inputs in males compared to hermaphrodites

Schematic diagram of the connectivity of the AVG neuron in both sexes in the adult stage based on electron microscopy reconstructions[2,3]. Chemical and electrical synapses between sensory (triangles), inter- (hexagons) and motor (circles) neurons are depicted as arrows and inhibitory

arrows, accordingly. Arrow thickness correlates with the degree of connectivity (number of sections over which *en passant* synapses are observed). Orange- hermaphrodites, cyan- males.

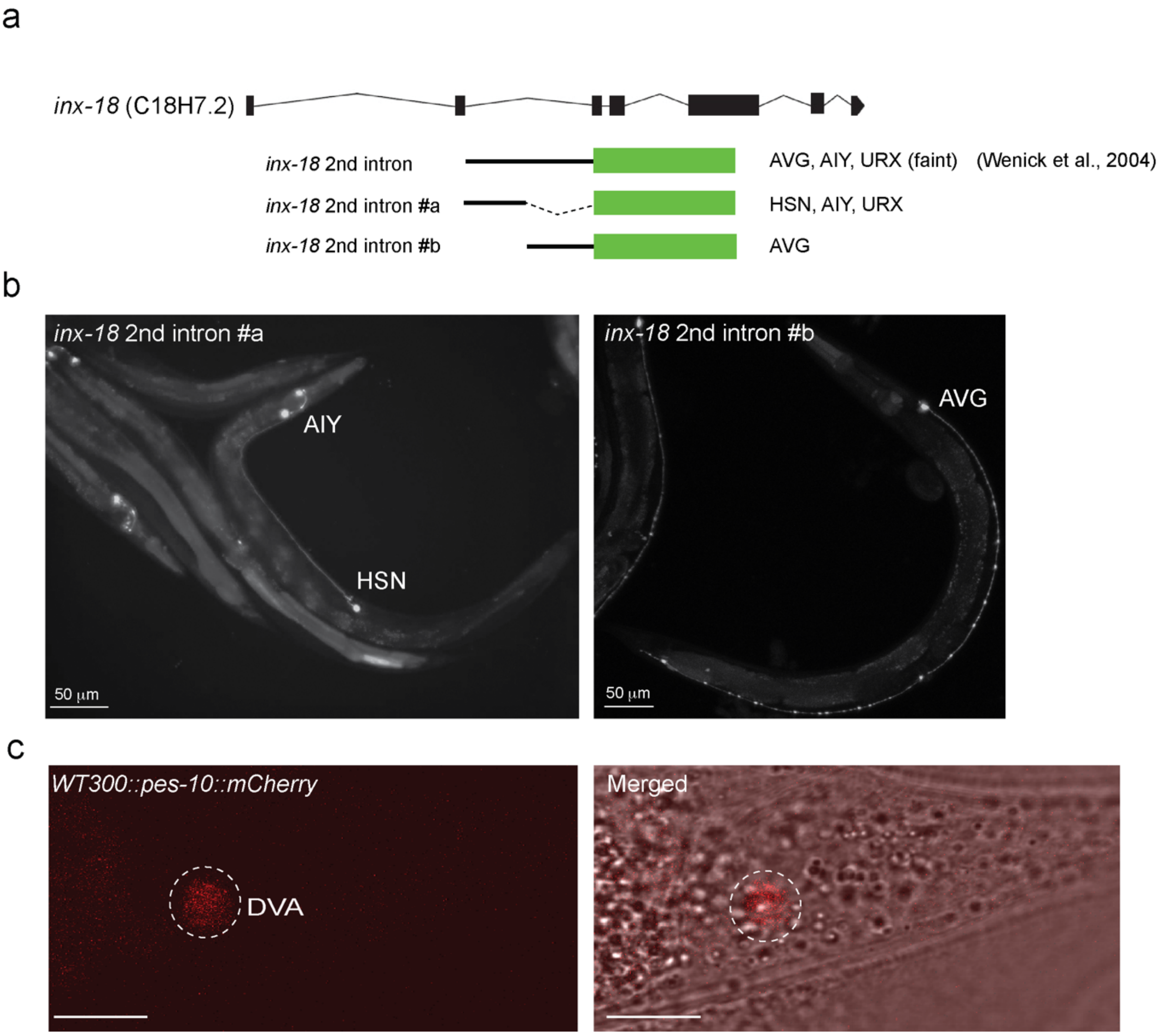

Supplementary Fig. 3: Cell-specific drivers in the sex-shared interneurons AVG and DVA

a Schematic of the bashing of the *inx-18* second intron[4] to drive specific expression in AVG. Identified neurons in which expression was observed are written to the right of each construct. **b** Representative confocal micrograph of *inx-18#a* (left) and *inx-18#b* (right). Scale bars are 50 µm. n=20 animals were examined for expression. **c** Representative confocal micrograph of DVA interneuron identified by the expression of *WT300::pes-10::mCherry*[5]. n=10 animals were examined for expression. Scale bars are 10 µm.

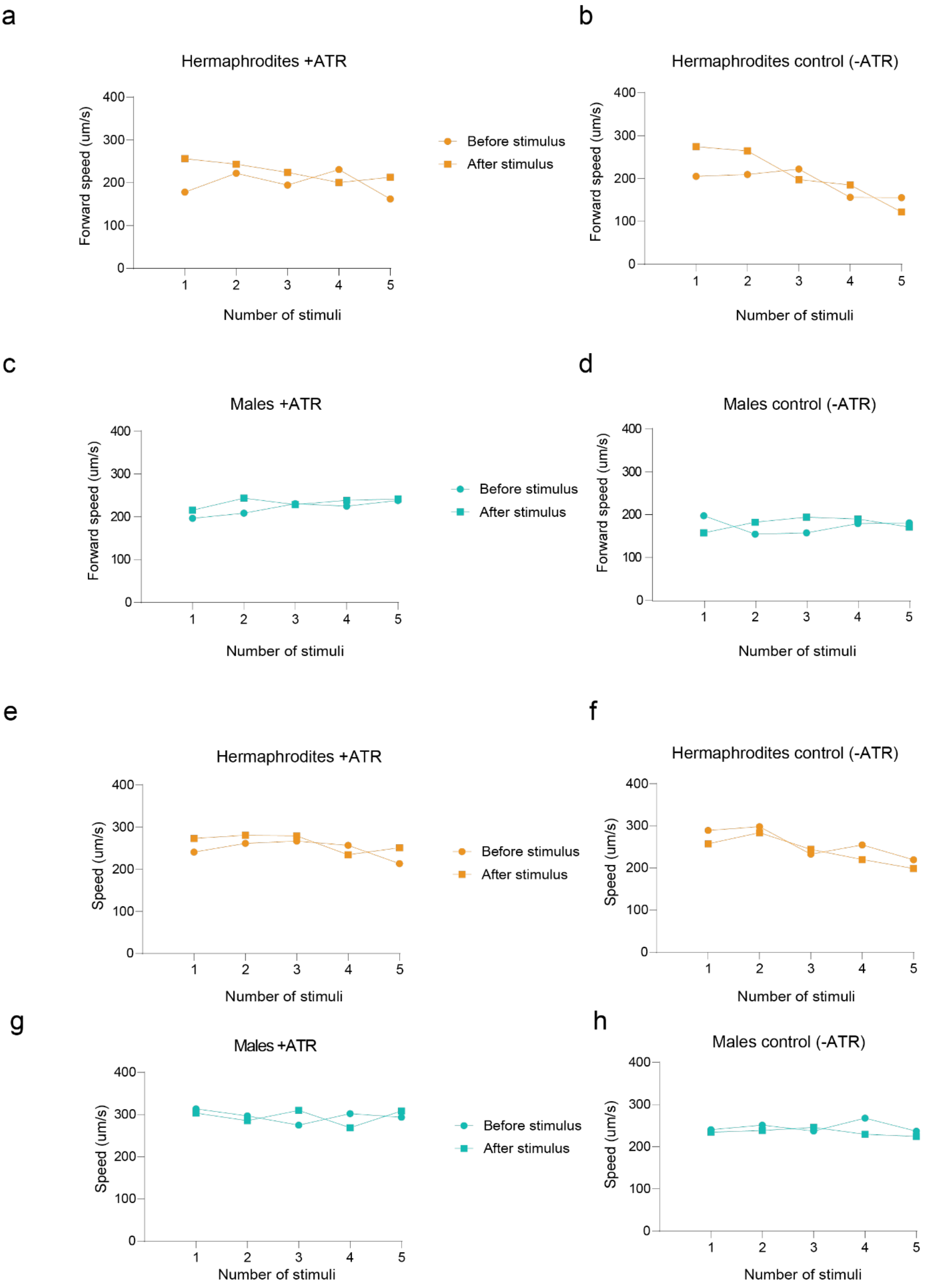

Supplementary Fig. 4: Optogenetic activation of AVG does not affect the locomotion of both sexes

The average forward speed (**a-b**) and total speed (forward and reverse, **e-f**) of hermaphrodites grown on ATR (n=21) (**a, e**) or control (n=20) (**b, f**) plates before and after each stimulus in a sequence of five stimuli (see *Methods*). The average forward speed (**c-d**) and total speed (forward and reverse, **g-h**) of males grown on ATR (n=12) (**c, g**) or control (n=17) (**d, h**) plates before and after each stimulus in a sequence of five stimuli (see *Methods*). Orange- hermaphrodites, cyan- males.

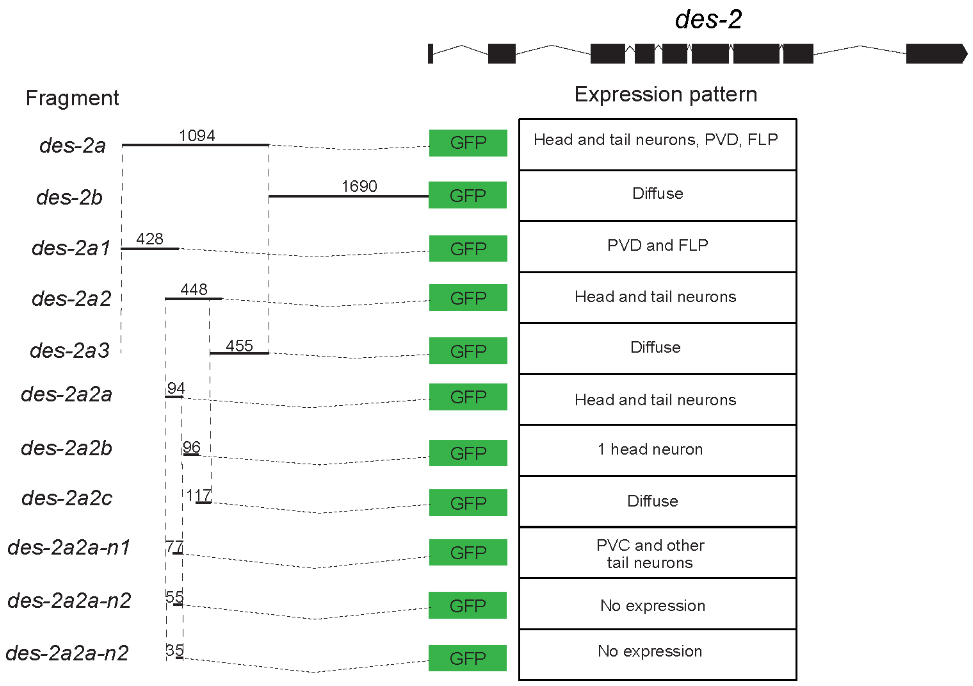

Supplementary Fig. 5: *des-2* promoter analysis

Schematic of the *des-2* promoter bashing. The fragments were fused to GFP and their expression patterns were examined under confocal microscopy.

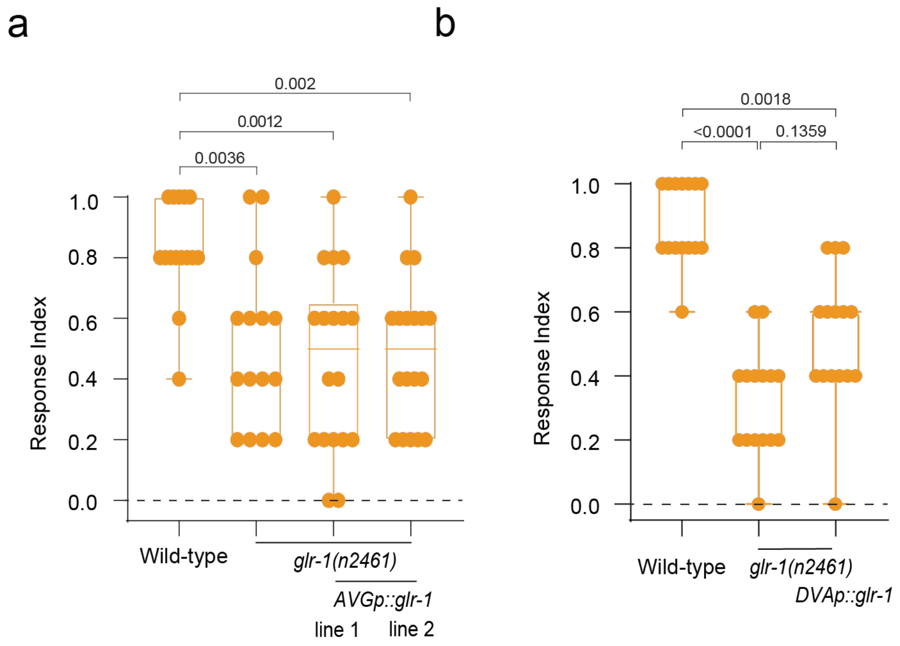

Supplementary Fig. 6: *glr-1* does not function through AVG or DVA to mediate tail mechanosensation in hermaphrodites

a Tail-touch responses of wild-type (n=14), *glr-1(n2461)* (n=14) and two extrachromosomal lines of *glr-1(n2461);AVGp::glr-1* (n=17 animals per group) hermaphrodites. **b** Tail-touch responses of wild-type, *glr-1(n2461)* and *glr-1(n2461);DVAp::glr-1* hermaphrodites. n=15 animals per group. The response index represents an average of the forward responses (scored as responded or not responded) in five assays for each animal. We performed a Kruskal-Wallis test followed by a Dunn's multiple comparison test. All Bar graphs are a box-and-whiskers type of graph, min to max showing all points. The vertical bars represent the median.

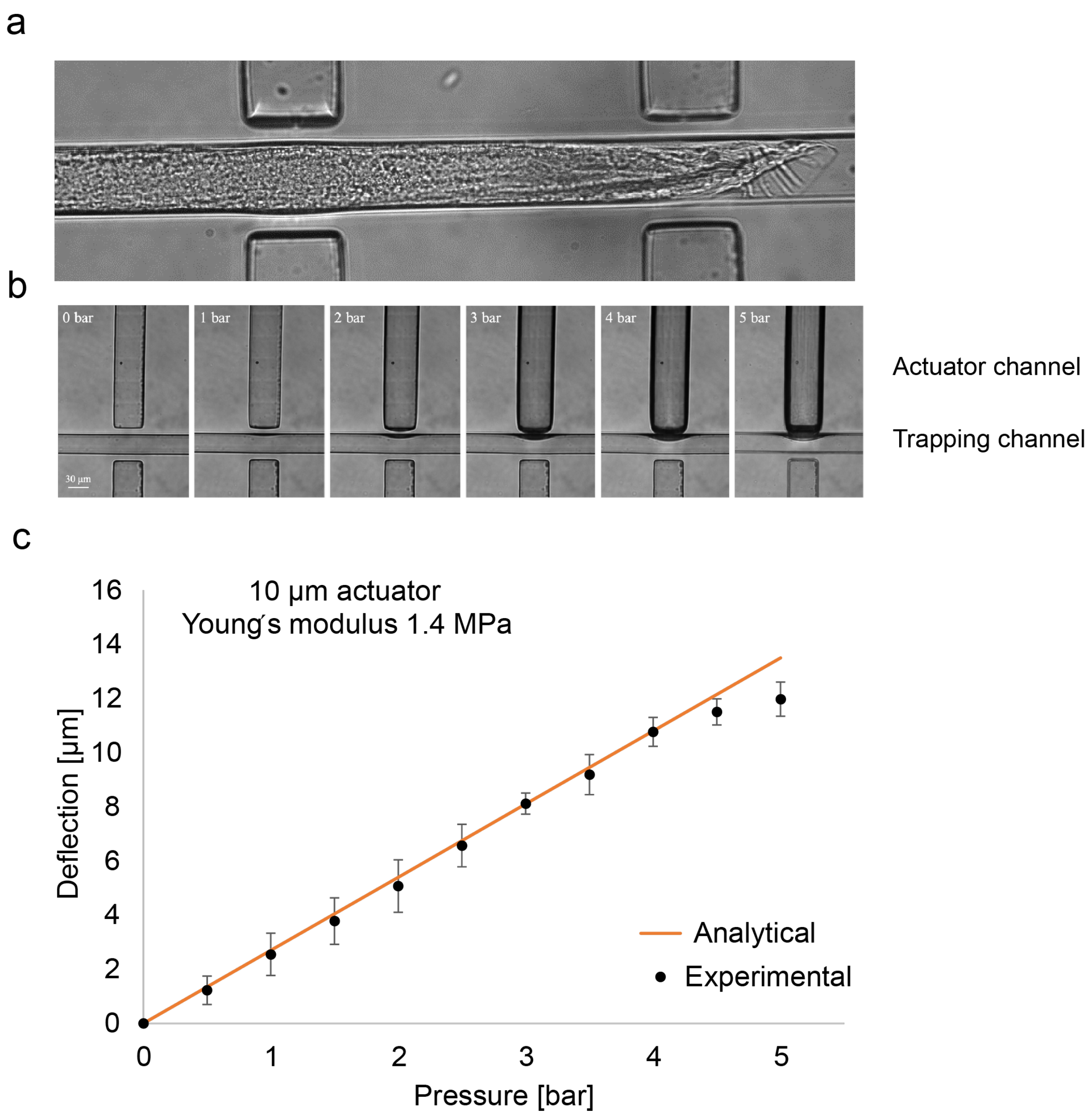

Supplementary Fig. 7: Calibration of the microfluidic device designed to measure calcium activity traces in response to tail mechanical stimulation

a The microscopic image of a male immobilized in a trapping channel. **b, c** Deflection of the actuator under different pressure values, from 0 to 5 bar. Deflection was measured using ImageJ without the presence of an animal inside the trapping channel. n=4 membranes; Mean +/- standard deviation.

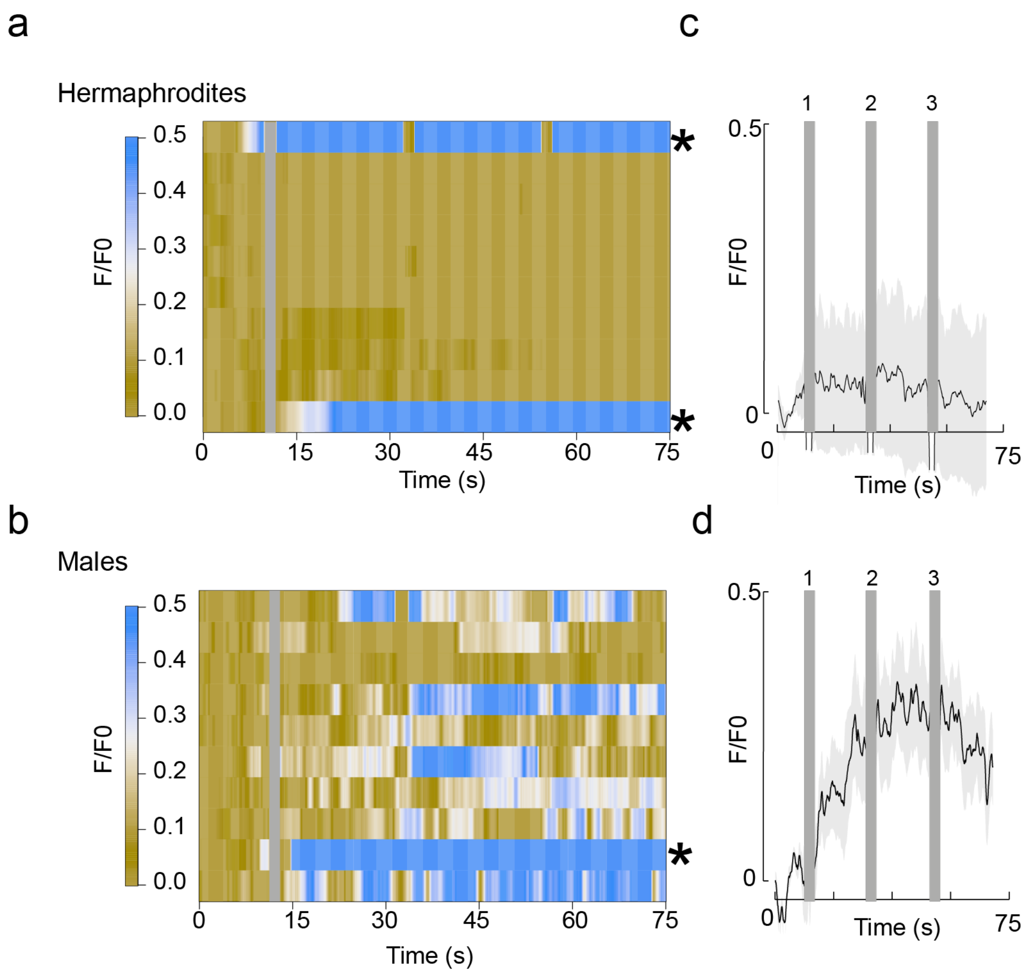

Supplementary Fig. 8: AVG neuronal recordings suggest light-evoked activity

AVG GCaMP6s calcium responses to three consecutive tail mechanical stimulations of hermaphrodites (**a**) and males (**b**). Heatmaps represent the calcium levels of individual worms. Asterisks represent individual worms where light-evoked activity is suspected to occur. Gray vertical line represents the times when the first stimulus was applied. Average and SEM traces of AVG calcium responses of hermaphrodites (**c**) and males (**d**). Gray vertical lines represent the times when a stimulus was applied. n = 10 animals per group. Each stimulus lasted two seconds (see *Methods*).

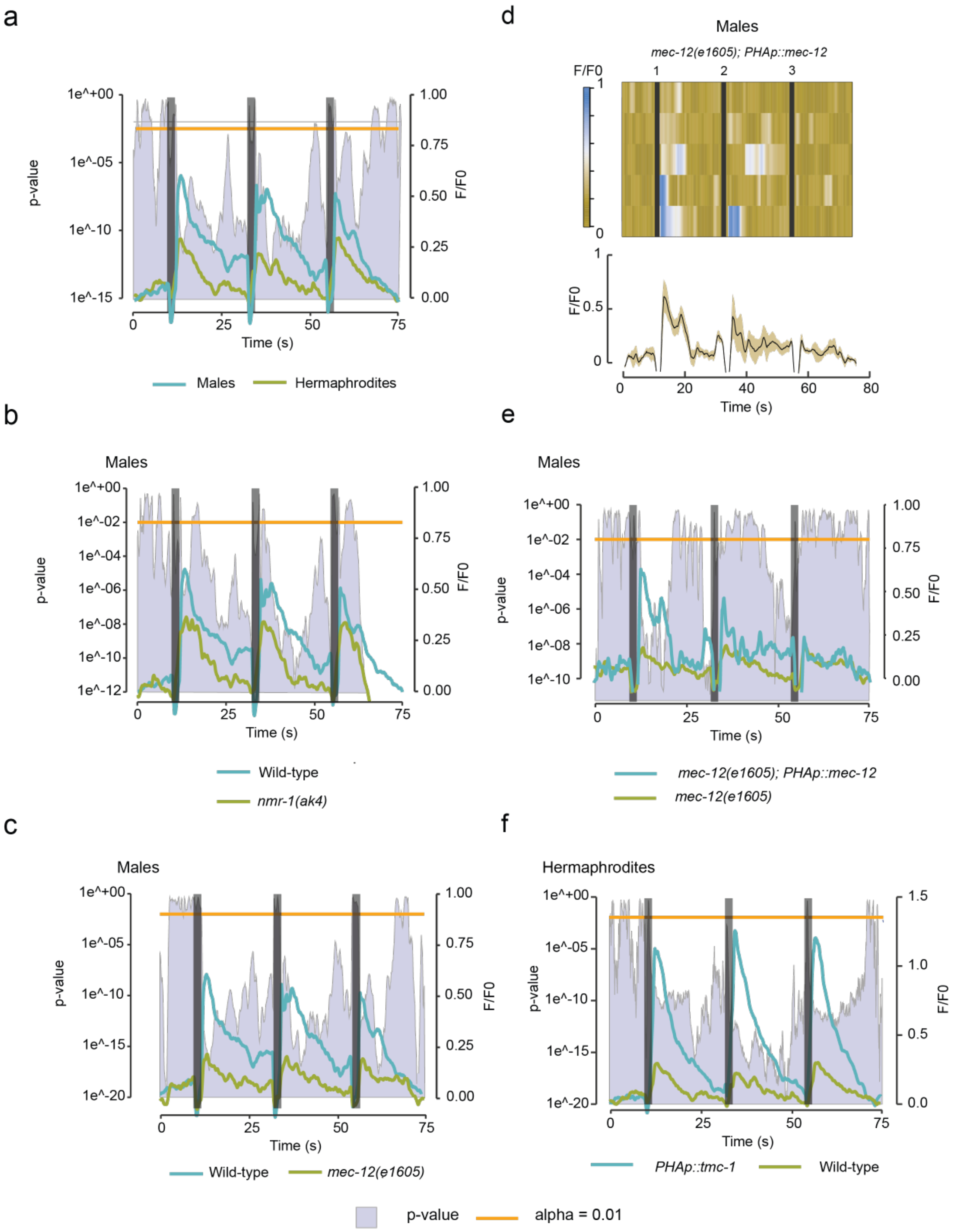

Supplementary Fig. 9: Statistical representation of calcium activity traces in AVG under different conditions

a The average calcium responses of males and hermaphrodites (smoothed and corrected, see *Methods*) to the running t-value (one-sided t-test) in each time point (blue shaded curve). Gray vertical lines represent the times when a stimulus was applied. Orange line represents p-value = 0.01. **b, c** The average calcium responses of wild-type and *nmr-1(ak4)* males (**b**) and wild-

type and *mec-12(e1605)* males (**c**) (smoothed and corrected, see *Methods*) in relation to the running t-value (one-sided t-test) in each time point (blue shaded curve). Gray vertical lines represent the times when a stimulus was applied. Orange line represents p-value = 0.01. **d** AVG GCaMP6s calcium responses extracted from the axons of *mec-12(e1605)* males with *PHAp::mec-12* to three consecutive tail mechanical stimulations. Stacked kymographs represent the GCaMP intensity vs. time of individual recordings. Graphs represent average and SD traces of AVG calcium responses. Black vertical lines represent the time when a stimulus was applied. n = 5 animals. **e** The average calcium responses of *mec-12(e1605)* males and *mec-12(e1605)* males with *PHAp::mec-12* transgene (smoothed and corrected, see *Methods*) to the running t-value (one-sided t-test) in each time point (blue shaded curve). Gray vertical lines represent the times when a stimulus was applied. Orange line represents p-value = 0.01. **f** The average calcium responses of wild-type hermaphrodites and hermaphrodites with *PHAp::tmc-1* transgene (smoothed and corrected, see *Methods*) to the running t-value (one-sided t-test) in each time point (blue shaded curve). Gray vertical lines represent the times when a stimulus was applied. Orange line represents p-value = 0.01.

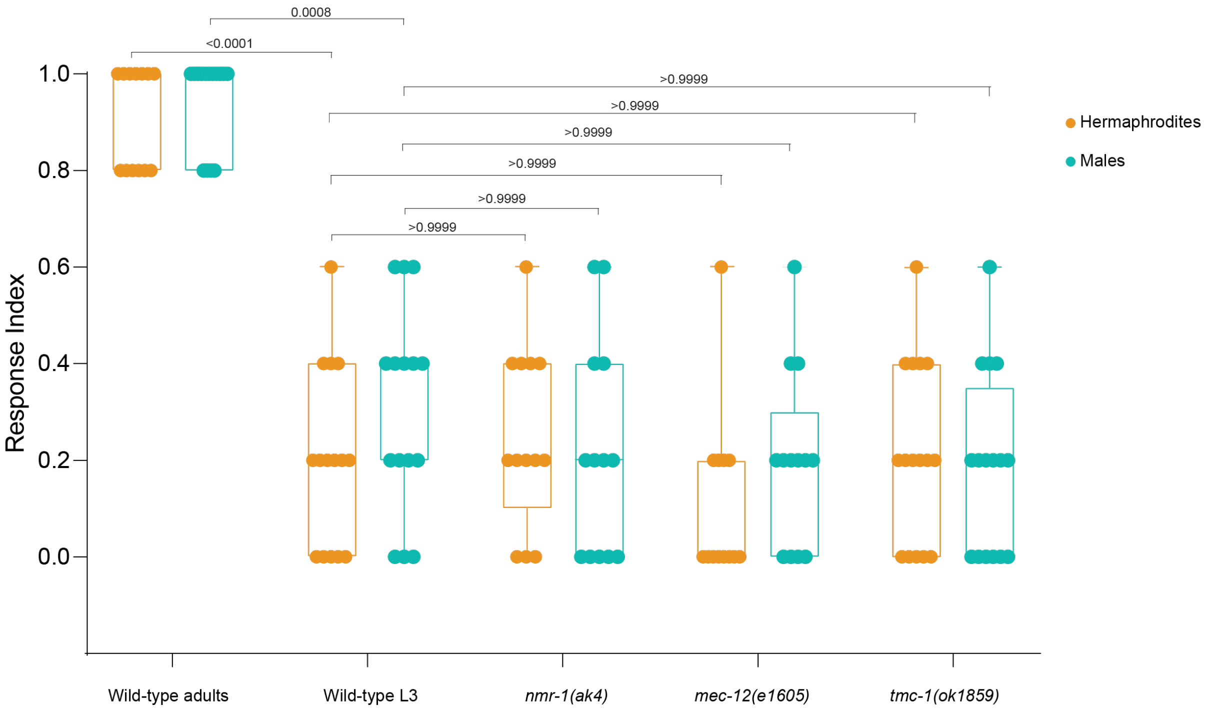

Supplementary Fig. 10: Juvenile animals show low tail-touch responses in both sexes

Tail-touch responses of adult animals in wild-type (n=15 animals per group) and of juvenile animals in wild-type (n=15 animals per group), *nmr-1(ak4)* (n=13 animals per group)*, mec-12(e1605)* (n=13 animals per group) and *tmc-1(ok1859)* (n=16 animals per group) of both hermaphrodites and males. We performed a Kruskal-Wallis test followed by a Dunn's multiple comparison test. Bar graph is a box-and-whiskers type of graph, min to max showing all points. The vertical bars represent the median.

Supplementary Table 1: List of strains used in this study

Strain	Description	Source
CX14373	*kyEx4571 [pNP403 (tag-168::HisCl1::SL2::GFP 5 [ng/µL); myo-3::mCherry 10 ng/µL*	*Caenorhabditis Genetics Center*
OH16196	*otEx7437[srg-13::HisCl::SL2::GFP 50ng/ul, ttx-3::mCherry 50ng/ul]; him-5 (e1490) V*	Oliver Hobert's lab
OH13689	*otEx6341[gpa-6::HisCl1::SL2::GFP 50ng/ul; ttx-3:cherry 50ng/ul]; him-5(e1490) V*	[6]
OH14826	*otEx6906 [eat-4p11del11:::HisCl::SL2::GFP 50ng/µl; unc-122::gfp 50ng/µl]; him-5(e1490) V*	[1]
CB4088	*him-5(e1490) V*	*Caenorhabditis Genetics Center*
CB3284	*mec-12(e1605) III*	*Caenorhabditis Genetics Center*
MOS463	*mec-12(e1605) III; him-5(e1490) V*	This study
RB1546	*T13G4.3(ok1859) X*	*Caenorhabditis Genetics Center*
MOS478	*T13G4.3(ok1859) X; him-5(e1490) V*	This study
CX4544	*ocr-2(ak47) IV*	*Caenorhabditis Genetics Center*
MOS325	*ocr-2(ak47) IV; him-5(e1490) V*	This study
CX10	*osm-9(ky10) IV*	*Caenorhabditis Genetics Center*
MOS332	*osm-9(ky10) IV; him-5(e1490) V*	This study
MOS579	*mec-12(e1605) III; etyEx207[srg-13p::mec-12 30ng/ul; ttx-3::gfp 30ng/ul; pBS 40ng/ul]; him-5(e1490)*	This study
MOS580	*mec-12(e1605) III; etyEx207[gpa-6::mec-12 30ng/ul; ttx-3::gfp 30ng/ul; pBS 40ng/ul]; him-5(e1490)*	This study
MOS591	*T13G4.3(ok1859) X; etyEx215[srg-13p::mec-12 30ng/ul; ttx-3::gfp 30ng/ul; pBS 40ng/ul]; him-5(e1490)*	This study
MOS505	*etyEx174[eat-4p11::tmc-1 40ng/ul, ttx-3::gfp 30 ng/ul, pBS 30 ng/ul], T13G4.3(ok1859) X; him-5(e1490)*	This study
MOS182	*etyEx188[inx-18::HisCl1::SL2::GFP 50ng/ul; ttx-3:cherry 50ng/ul]; him-5(e1490) V*	This Study
MOS473	*etyEx159[WT300::pes-10::HisCl1::SL2::GFP 50ng/ul, ttx-3::gfp 30ng/ul; pBS 20ng/u;];him-5(e1490) V*	This study

MOS247	*otEx6335[inx-18::tra-2(ic)::SL2::2XNLS::tag-RFP 10ng/ul, ttx-3::GFP 50ng/ul, pBS 40ng/ul]; etyEx188[inx-18::HisCl1::SL2::GFP 50ng/ul; ttx-3:cherry 50ng/ul]; him-5(e1490)*	This study
MOS254	*otIs606[inx-18p::FEM-3::SL2::wcherry; pha-1+]; etyEx188[inx-18::HisCl1::SL2::GFP 50ng/ul; ttx-3:cherry 50ng/ul]; him-5(e1490)*	This study
MOS195	*etyEx44[inx-18delAIY::NpHR::mCherry 100 ng/ul + ttx-3::GFP 30 ng/ul]*	This study
SRS167	*pha-1(e2123) III; lite-1(ce314) X*	Alon Zaslaver's lab
MOS287	*him-5(e1490)V; lite-1(ce314) X*	This study
MOS203	*etyEx44[inx-18delAIY::NpHR::mCherry 100 ng/ul ttx-3::GFP 30 ng/ul +]; him-5(e1490)*	This Study
KP4	*glr-1(n2461) III*	*Caenorhabditis Genetics Center*
MOS340	*glr-1(n2461) III; him-5(e1490)*	This study
RB1808	*glr-2(ok2342) III*	*Caenorhabditis Genetics Center*
MOS326	*glr-2(ok2342) III. ; him-5(e1490) V*	This study
VM487	*nmr-1(ak4) II*	*Caenorhabditis Genetics Center*
MOS351	*nmr-1(ak4) II; him-5(e1490)*	This study
CB1489	*him-8(e1489) IV*	*Caenorhabditis Genetics Center*
VC2623	*nmr-2(ok3324) V*	*Caenorhabditis Genetics Center*
MOS346	*nmr-2(ok3324) V; him-8(e1489) IV*	This study
MOS434	*etyEx137[inx-18p::nmr-1 25ng/ul; ttx-3::gfp 20 ;ng/ul; pBS 55ng/ul]; nmr-1(ak4) II; him-5(e1490) V*	This study
MOS70	*otIs460 [inx-18p::wcherry; pha-1(+)]; him-8(e1489) IV*	Oliver Hobert's lab
MOS632	*etyEx181 [MVC11 15ng/ul, pRF4 50ng/ul, nmr-1 fosmid 15ng/ul, pBS 20ng/ul];otIs460 (inx-18p::wcherry; pha-1(+)); him-8(e1489) IV*	This study
MOS239	*otIs606 [inx-18p::FEM-3::SL2::wcherry; pha-1+]; him-5(e1490) V*	This study
MOS368	*otIs606 [inx-18p::FEM-3::SL2::wcherry; pha-1+]; nmr-1(ak4) II; him-5(e1490) V*	This study
MOS433	*etyEx142[pMO32(inx-18::tra-2(ic)::SL2::2NLS) 40 ng/ul; ttx-3::gfp 30 ng/ul, pBS 30 ng/ul]*	This study

MOS496	*etyEx142[pMO32(inx-18::tra-2(ic)::SL2::2NLS) 40 ng/ul; ttx-3::gfp 30 ng/ul, pBS 30 ng/ul]; him-5(e1490)*	This study
MOS506	*etyEx142[pMO32(inx-18::tra-2(ic)::SL2::2NLS) ng/ul; ttx-3::gfp 30 ng/ul, pBS 30 ng/ul]; nmr- 40 1(ak4) II; him-5(e1490)*	This study
MOS568	*otIs606 (inx-18p::FEM-3::SL2::wcherry; pha-1+)him-5(e1490);nmr-1(ak4) II otEx7437 Ex[srg-13::HisCl::GFP, ttx-3::mCherry]*	This study
MOS564	*otIs606 (inx-18p::FEM-3::SL2::wcherry; pha-1+)him-5(e1490);nmr-1(ak4) II;otEx6906 (eat-4p11del11:::HisCl::gfp 50ng/µl; unc-122::gfp 50ng/µl)*	This study
MOS570	*etyEx142[pMO32(inx-18::tra-2(ic)::SL2::2NLS) 40 ng/ul; ttx-3::gfp 30 ng/ul, pBS 30 ng/ul];him-5(e1490);nmr-1(ak4) II otEx7437 Ex[srg-13::HisCl::GFP, ttx-3::mCherry]*	This study
MOS566	*etyEx142[pMO32(inx-18::tra-2(ic)::SL2::2NLS) 40 ng/ul; ttx-3::gfp 30 ng/ul, pBS 30 ng/ul]; him-5(e1490);nmr-1(ak4) II;otEx6906 (eat-4p11del11:::HisCl::gfp 50ng/µl; unc-122::gfp 50ng/µl)*	This study
MOS495	*etyEx168[WT300::pes-10::nmr-1 25ng/ul, ttx-3::gfp 20 ng/ul, pBS 55 ng/ul]; nmr-1(ak4) II; him-5(e1490)*	This study
MOS480	*etyEx31[inx-18b::GCaMP6s 30ng/ul, sra-6::WrmScarlet 30ng/ul, PHB::mCherry MVC15 40ng/ul]; lite-1(ce314) X; him-5(e1490)*	This study
MOS488	*etyEx31[inx-18b::GCaMP6s 30ng/ul, sra-6::WrmScarlet 30ng/ul, PHB::mCherry MVC15 40ng/ul]; lite-1(ce314) X; nmr-1(ak4) II; him-5(e1490)*	This study
MOS510	*etyEx31[inx-18b::GCaMP6s 30ng/ul, sra-6::WrmScarlet 30ng/ul, PHB::mCherry MVC15 40ng/ul]; lite-1(ce314) X; nmr-1(ak4) II; mec-12(e1605) III; him-5(e1490)*	This study
MOS621	*etyEx233[srg-13p::tmc-1 30 ng/ul; pBS 60 ng/ul; unc-122::gfp 10 ng/ul]; him-5; etyEx31 Ex[inx-18b::GCaMP6s, sra-6::WrmScarlet, PHB::mCherry MVC15];lite-1(ce314) X*	This study
MOS629	*etyEx238[srg-13p::tmc-1 30 ng/ul; pBS 40 ng/ul; ttx-3::gfp 30 ng/ul];T13G4.3(ok1859) X; him-5(e1490)*	This study

DA509	*unc-31(e928) IV*	*Caenorhabditis Genetics Center*
MOS588	*nmr-1(ak4) II; mec-12(e1605) III; him-5(e1490)*	This study
MOS477	*mec-12(e1605) III; etyEx161[che-12p::mec-12 30ng/ul; ttx-3::gfp 20ng/ul; pBS 50ng/ul]; him-5(e1490) V)*	This study
BC10751	*dpy-5(e907) I; sEx10751*	*Caenorhabditis Genetics Center*
MOS481	*dpy-5(e907) I; sEx10751; him-5(e1490) V*	This study
MOS583	*etyEx211[genomic mec-12::GFP PCR fusion 1 ng/ul; ttx-3::mCherry 30 ng/ul; pBS 70 ng/ul];him-5(e1490) V*	
AQ4330	*ljEx1221[Ptmc-1(4kb)::mKate2]*	[7]
OH12503	*otIs520[eat-4p11::gfp;ttx-3::cherry]; him-5(e1490) V*	Oliver Hobert's lab
MOS511	*ljEx1221[Ptmc-1(4kb)::mKate2]; otIs520(eat-4p11::gfp;ttx-3::cherry); him-5(e1490)*	This study
MOS533	*etyEx186[inx-18#b::GFP 50ng/ul, pha-1(+) 50ng/ul], pha-1(e2123)*	This study
MOS534	*etyEx187[inx-18#a::GFP 50ng/ul, pha-1(+) 50ng/ul], pha-1(e2123)*	This study
MOS466	*etyEx156[pHS11(WT300::pes-10::mCherry) 40ng/ul; myo-2::mCherry 5ng/ul; pBS 55ng/ul]*	This study
MOS214	*etyEx54[inx-18delAIY::Chr2::mCherry 50 ng/ul + ttx-3::GFP 30 ng/ul + pBS 20 ng/ul]*	This study
MOS283	*etyEx54[inx-18delAIY::Chr2::mCherry 50 ng/ul + ttx-3::GFP 30 ng/ul + pBS 20 ng/ul];him-5(e1490)V; lite-1(ce314)*	This Study
MOS452	*etyEx148(pMO41(inx-18p::GLR-1::GFP) 40 ng/ul, ttx-3::gfp 30 ng/ul, pBS 30 ng/ul); glr-1(n2461) III; him-5(e1490) V*	This study
MOS453	*,etyEx149[pMO41(inx-18p::GLR-1::GFP 40ng/ul) ttx-3::gfp 30ng/ul, pBS 30ng/ul]; glr-1(n2461) III; hin-5(e1490)*	This study
MOS577	*etyEx205[WT300::pes-10::glr-1 30 ng/ul; ttx-3::gfp 30 ng/ul; pBS 40 ng/ul];glr-1(n2461);him-5(e1490) V*	This study
MOS118	*etyEx31[inx-18b::GCaMP6s 30ng/ul, sra-6::WrmScarlet 30ng/ul, PHB::mCherry MVC15 40ng/ul]; him-5(e1490)*	[8]

MOS581	*him-5(e1490); etyEx31 Ex[inx-18b::GCaMP6s, sra-6::WrmScarlet, PHB::mCherry MVC15];lite-1(ce314) X;mec-12(e1605) III.; etyEx209[srg-13::mec-12 30 ng/ul; ttx03::gfp 30 ng/ul;pBS 40 ng/ul]*	This study

Supplementary References

1. Serrano-Saiz, E., Oren-Suissa, M., Bayer, E. A. & Hobert, O. Sexually Dimorphic Differentiation of a C. elegans Hub Neuron Is Cell Autonomously Controlled by a Conserved Transcription Factor. *Curr Biol* 27, 199–209 (2017).

2. Cook, S. J. *et al.* Whole-animal connectomes of both Caenorhabditis elegans sexes. *Nature* 571, 63–71 (2019).

3. White, J. G., Southgate, E., Thomson, J. N. & Brenner, S. The structure of the nervous system of Caenorhabditis elegans. *Philos. Trans. R. Soc. Lond. B Biol. Sci* 314, 1–340 (1986).

4. Wenick, A. S. & Hobert, O. Genomic cis-regulatory architecture and trans-acting regulators of a single interneuron-specific gene battery in C. elegans. *DEVCEL* 6, 757–770 (2004).

5. Robinson, C. P., Schwarz, E. M. & Sternberg, P. W. Identification of DVA Interneuron Regulatory Sequences in Caenorhabditis elegans. *Plos One* 8, e54971 (2013).

6. Oren-Suissa, M., Bayer, E. A. & Hobert, O. Sex-specific pruning of neuronal synapses in Caenorhabditis elegans. *Nature* 533, 206–211 (2016).

7. Kaulich, E., Walker, D. S., Tang, Y.-Q. & Schafer, W. R. The Caenorhabditis elegans tmc-1 is involved in egg-laying inhibition in response to harsh touch. *Micropublication Biology* 2021, 10.17912/micropub.biology.000439 (2021).

8. Salzberg, Y. *et al.* Synaptic Protein Degradation Controls Sexually Dimorphic Circuits through Regulation of DCC/UNC-40. *Curr Biol* 30, 4128-4141.e5 (2020).
