# Supplementary material for: Sexually dimorphic architecture and function of a mechanosensory circuit in C. elegans
Source: Nat Commun. 2022 Nov 11;13:6825. doi: 10.1038/s41467-022-34661-3 (PMC9652301; doi:10.1038/s41467-022-34661-3)
Supplement: Supplementary file 3 — Description of Additional Supplementary Files [file 41467_2022_34661_MOESM3_ESM.pdf]

Description of Additional Supplementary Files

File Name: Supplementary Movie 1
Description: Representative video of a *lite-1(ce314)* mutant male expressing GCaMP6s in AVG, trapped inside the microfluidic device and subjected to three consecutive stimulations to the tail at the indicated time points. Only the anterior part of the animal is shown. Scale bar is 50µm. Neurons in the head are bleed-through due to the co-injection of the marker *sra-6::WrmScarlet*.

File Name: Supplementary Data 1
Description: Autocad file with microfluidic design for mechanical stimulation to fit the size of males.
