# Supplementary material for: Sexually dimorphic architecture and function of a mechanosensory circuit in C. elegans
Source: Nat Commun. 2022 Nov 11;13:6825. doi: 10.1038/s41467-022-34661-3 (PMC9652301; doi:10.1038/s41467-022-34661-3)
Supplement: Supplementary file 6 — Reporting Summary [file 41467_2022_34661_MOESM6_ESM.pdf]

nature portfolio

Corresponding author(s): Oren-Suissa Meital

Last updated by author(s): Oct 27, 2022

Reporting Summary

Nature Portfolio wishes to improve the reproducibility of the work that we publish. This form provides structure for consistency and transparency in reporting. For further information on Nature Portfolio policies, see our Editorial Policies and the Editorial Policy Checklist.

Statistics

For all statistical analyses, confirm that the following items are present in the figure legend, table legend, main text, or Methods section.

n/a	Confirmed	
☐	☒	The exact sample size (*n*) for each experimental group/condition, given as a discrete number and unit of measurement
☒	☐	A statement on whether measurements were taken from distinct samples or whether the same sample was measured repeatedly
☐	☒	The statistical test(s) used AND whether they are one- or two-sided<br>*Only common tests should be described solely by name; describe more complex techniques in the Methods section.*
☒	☐	A description of all covariates tested
☐	☒	A description of any assumptions or corrections, such as tests of normality and adjustment for multiple comparisons
☐	☒	A full description of the statistical parameters including central tendency (e.g. means) or other basic estimates (e.g. regression coefficient) AND variation (e.g. standard deviation) or associated estimates of uncertainty (e.g. confidence intervals)
☐	☒	For null hypothesis testing, the test statistic (e.g. *F*, *t*, *r*) with confidence intervals, effect sizes, degrees of freedom and *P* value noted<br>*Give P values as exact values whenever suitable.*
☒	☐	For Bayesian analysis, information on the choice of priors and Markov chain Monte Carlo settings
☒	☐	For hierarchical and complex designs, identification of the appropriate level for tests and full reporting of outcomes
☒	☐	Estimates of effect sizes (e.g. Cohen's *d*, Pearson's *r*), indicating how they were calculated

Our web collection on statistics for biologists contains articles on many of the points above.

Software and code

Policy information about availability of computer code

Data collection	Zen (Zeiss, v2.3), Wormlab (MBF Biosciences)
Data analysis	ImageJ (version 1.52p), Zen (Zeiss, v2.3), Wormlab (MBF Biosciences), Prism 9 (GraphPad), Illustrator V 25.0.1, Photoshop V 22.0.1, MATLAB (vR2019B), R (V1.3-1), Python (Version 2.7), IgroPro (Version 6.370). Original code has been deposited at https://doi.org/10.34933/wis.000652 and is publicly available as of the date of publication.

For manuscripts utilizing custom algorithms or software that are central to the research but not yet described in published literature, software must be made available to editors and reviewers. We strongly encourage code deposition in a community repository (e.g. GitHub). See the Nature Portfolio guidelines for submitting code & software for further information.

Data

Policy information about availability of data

All manuscripts must include a data availability statement. This statement should provide the following information, where applicable:

- Accession codes, unique identifiers, or web links for publicly available datasets
- A description of any restrictions on data availability
- For clinical datasets or third party data, please ensure that the statement adheres to our policy

The authors declare that all data generated or analyzed during this study are included in this published article (and its supplementary information files). The raw data generated in this study are provided in the Source Data file.

Human research participants

Policy information about studies involving human research participants and Sex and Gender in Research.

Reporting on sex and gender	N/A
Population characteristics	N/A
Recruitment	N/A
Ethics oversight	N/A

Note that full information on the approval of the study protocol must also be provided in the manuscript.

Field-specific reporting

Please select the one below that is the best fit for your research. If you are not sure, read the appropriate sections before making your selection.

☒ Life sciences ☐ Behavioural & social sciences ☐ Ecological, evolutionary & environmental sciences

For a reference copy of the document with all sections, see nature.com/documents/nr-reporting-summary-flat.pdf

Life sciences study design

All studies must disclose on these points even when the disclosure is negative.

Sample size	Wherever possible, animal numbers were kept at 15-20 per sex/genotype. Sample size was determined to be no less than 10 animals per group. For our assays, the sample size that is desired for observing a phenotype is 12-15 animals per group. When genetic backgrounds are challenging for growth and maintenance, we used a sample size lower than 15 but not lower than 10.
Data exclusions	No data were excluded
Replication	Wild-type and untreated controls were done separatly for each experiment and as such independently confirm the same findings and can be consisered as replicates. For RNAi, positive results were rpeated with mutant animals. For all experiments, mutant positive-phenotypes were repeated at least 3 times independently.
Randomization	Animals for each experiment were picked randomly from large population plates.
Blinding	Wherever possible, the experimenter was blinded to the genotype of the animals tested- Figure 1e, Figure 2b-c, Figure 3f-g, Figure 4a,d,e,g, Figure 6a-b. Figure 7 was analyzed blindly. For obvious reasons, the sex of the animals tested was known to the experimenter. Most behavioral assays were done blinded except in times where the group tested was known to the experimenter for different reasons (number of animals, conditions of experiment). Examples include histamine-based assays and RNAi-based assays where the conditions of experiment involve specific plates that are known to the experimenter.

Reporting for specific materials, systems and methods

We require information from authors about some types of materials, experimental systems and methods used in many studies. Here, indicate whether each material, system or method listed is relevant to your study. If you are not sure if a list item applies to your research, read the appropriate section before selecting a response.

Materials & experimental systems

n/a	Involved in the study	
☒	☐	Antibodies
☒	☐	Eukaryotic cell lines
☒	☐	Palaeontology and archaeology
☐	☒	Animals and other organisms
☒	☐	Clinical data
☒	☐	Dual use research of concern

Methods

n/a	Involved in the study	
☒	☐	ChIP-seq
☒	☐	Flow cytometry
☒	☐	MRI-based neuroimaging

Animals and other research organisms

Policy information about studies involving animals; ARRIVE guidelines recommended for reporting animal research, and Sex and Gender in Research

Laboratory animals	C. elegans, N2 bristol strain, hermaphrodites and males of various developmental stages. All the strains used in this study are listed in the supplementary material file. Most experiments were performed at 1 day adult stage, except behavioral assay for supplementary fig.10 that was done at L3 stage when specified.
Wild animals	none
Reporting on sex	Sexual identity was considered and fully reported.
Field-collected samples	No field collected samples were used in the study
Ethics oversight	Ethical approval is not required for work with soil nematodes

Note that full information on the approval of the study protocol must also be provided in the manuscript.
